# LAPAROSCOPIC RESECTION OF GASTROINTESTINAL STROMAL TUMORS (GIST)

**DOI:** 10.1590/0102-6720201600010001

**Published:** 2016

**Authors:** Marcelo de Paula LOUREIRO, Rômulo Augusto Andrade de ALMEIDA, Christiano Marlo Paggi CLAUS, Eduardo Aimoré BONIN, Antônio Moris CURY-FILHO,, Daniellson DIMBARRE, Marco Aurélio Raeder da COSTA, Marcílio Lisboa VITAL

**Affiliations:** 1 Jacques Perissat Institute; 2 Positivo University, Curitiba, PR, Brazil

**Keywords:** Laparoscopy, GIST, Surgery, Gastrectomy, Enterectomy

## Abstract

**Background:**

Gastrointestinal mesenchymal or stromal tumors (GIST) are lesions originated on digestive tract walls, which are treated by surgical resection. Several laparoscopic techniques, from gastrectomies to segmental resections, have been used successfully.

**Aim:**

Describe a single center experience on laparoscopic GIST resection.

**Method:**

Charts of 15 operated patients were retrospectively reviewed. Thirteen had gastric lesions, of which ten were sub epithelial, ranging from 2-8 cm; and three were pure exofitic growing lesions. The remaining two patients had small bowel lesions. Surgical laparoscopic treatment consisted of two distal gastrectomies, 11 wedge gastric resections and two segmental enterectomies. Mechanical suture was used in the majority of patients except on six, which underwent resection and closure using manual absorbable sutures. There were no conversions to open technique.

**Results:**

Mean operative time was 1h 29 min±92 (40-420 min). Average lenght of hospital stay was three days (2-6 days). There were no leaks, postoperative bleeding or need for reintervention. Mean postoperative follow-up was 38±17 months (6-60 months). Three patients underwent adjuvant Imatinib treatment, one for recurrence five months postoperatively and two for tumors with moderate risk for recurrence .

**Conclusion:**

Laparoscopic GIST resection, not only for small lesions but also for tumors above 5 cm, is safe and acceptable technique.

## Abstract (pt)

***Racional*:**

Os tumores estromais ou mesenquimais gastrointetinais (GIST) são lesões originárias da parede do tubo digestivo cujo tratamento requer remoção cirúrgica. Diversas técnicas por via laparoscópica - gastrectomias e ressecções segmentares - têm sido empregadas com sucesso.

***Objetivo*:**

Apresentar a experiência de um serviço de cirurgia com ressecção laparoscópica de GIST.

***Métodos*:**

Foram avaliados 15 pacientes com GIST operados revisados retrospectivamente. Treze tiveram lesões gástricas, das quais 10 eram subepiteliais entre 2-8 cm. Três eram lesões exofíticas puras. Dois apresentavam lesões no intestino delgado. O tratamento cirúrgico por laparoscopia consistiu de duas gastrectomias distais; 11 ressecções gástricas em cunha e duas enterectomias segmentares. Sutura mecânica foi utilizada na maioria dos doentes, exceto em seis com suturas absorvíveis manuais. Não houve conversões para laparotomia.

***Resultados*:**

O tempo médio das operações foi de 89±92 min (40-420). A hospitalização média foi de três dias (2-6). Não houve fístula, sangramento pós-operatório ou necessidade de reintervenção por complicação cirúrgica. O seguimento médio pós-operatorio foi de 38±17 meses (6-60). Três pacientes foram encaminhados para terapia adjuvante com mesilato de imatinib, um deles por recidiva precoce aos cinco meses, e os outros dois por apresentarem risco moderado para recidiva.

***Conclusão*:**

A ressecção laparoscópica de GIST, mesmo os maiores de 5 cm, é procedimento factível e seguro.

## INTRODUCTION

Gastrointestinal stromal or mesenquimal tumors (GIST) are lesions originated from digestive tube walls, which tend to occur in individuals older than 60 years. Its anual incidence is 10 cases per million inhabitants^12^^,^ 17. This entity derives from the Cajal's cell membrane receptor C-kit mutation. Cajal's cells are also known as intestinal pacemaker cells^15^. Such mutation has direct influence on proliferation and cell death. GIST definite diagnosis is only possible in the presence of CD117 (C-Kit) marker on immunohistochemistry, positive in over 95% of the cases^26^. GIST tumors are intramural growing lesions, which makes its biopsy very challenging. They usually present on a variable biological behavior, being benign in the majority of cases. Around 30% present as clinically malignant lesions^8^. Treatment is surgical complete resection. Neoadjuvant chemotherapy sometimes is indicated in the management of large lesions. Adjuvance is reserved for recurrence or unresectable metastasis^16^^,^ 24.

GISTs surgical resections can be performed either laparoscopically or as open conventional procedures^1^^,^ 28. There are no available randomized studies comparing the two approaches. Series of patients or case-control studies are, therefore, the best source to evaluate pros and cons of each technique. 

The aim of this study was to report a single center experience on laparoscopic GIST resection.

## METHODS

A retrospective non-comparative review of confirmed GIST cases was performed in a single center. Patients harboring submucosal gastric lesions larger than 2 cm were evaluated by endoscopic ultrasound when available, and then submitted to either resection or close follow up. Suspicious lesions on patients being considered for bariatric surgery were resected before or during the bariatric procedure according to tumor location. Non-gastric lesions were included in the review only if GIST diagnosis was confirmed.

All patients with primary gastric GISTs were initially prepared for laparoscopic resection. The resection techniques were chosen according to tumor location. Therefore local resections, wedge resections and partial gastrectomies were performed. Small bowel lesions were treated by segmental intestinal resections. Wedge resections were performed either with or without mechanical stapling devices, according to surgeon's preference. Laparoscopic access to cavity followed classical techniques previously described. Special care was taken in order to avoid tumor capsule rupture during specimen handling and extraction from the cavity. Specimen retrieval was performed through a special pouch (Endocatch, Covidien).

Baseline data including gender, age, body mass index (BMI), symptoms and signs, endoscopy and image findings, previous abdominal operations, operation time, length of stay, complications, re-interventions and readmissions on six months period were collected. Patients were classified according to prognostic criteria of risk related to recurrence and tumor death (Table 1). Operated patients were submitted to laboratory and clinical follow up. Patients with metastatic disease at time of resection and patients operated without histologic confirmation of GIST were excluded from the study.

**TABLE 1:** - Risk stratification for primary GIST by mitosis ratio, tumor size and location.

**Mitosis ratio**	**Tumor size**	Location risk*		
		**Gastric**	**Small Bowell**	**Rectum**
≤5 per HPF	≤2 cm	Very Low	Very Low	Very Low
	> 2cm and ≤5cm	Low	Low	Low
	> 5 cm and ≤10 cm	Low	Moderate	Insufficient Data
	>10 cm	-	High	High
>5 per HPF	≤2 cm	Low	High	High
	> 2 cm and ≤5 cm	Moderate	High	High
	> 5 cm and ≤10cm	High	High	Insifficient Data
	> 10 cm	High	High	High

HPF=high power field;*=defined by metastasis or tumor related death (Adapted from Miettinen&Lasota)

## RESULTS

Between January 2009 and October 2013, 15 patients with GIST diagnosis were operated in the Division of General Surgery, Jacques Perissat Institute, Curitiba, PR, Brazil. Patients characteristics are presented on Table 2.

**TABLE 2:** - Patient base characteristics and tumor location.

**Characteristics**	**GIST (n=15)**
Gender: female/male, n(%)	9 (66%)/6 (33%)
BMI mean±SD (variation), kg/m²	26±12 (20-40)
Tumor location gastric/intestinal n (%)	12 (80%) / 3 (20%)

BMI=body mass index

Seven patients (46%) were completely asymptomatic being diagnosed with GISTs on routine image exams, on preparatory workout or even during the operation for GERD disease or bariatric procedure. Among symptomatic individuals, two complained of melena, three epigastric pain, one dysphagia, one post-meal vomits and one tenesmus.

All patients, no matter location of the lesion, underwent an upper GI endoscopy. During the exam were identified 10 submucosal gastric lesions ranging from 2 to 8 cm, one of them presenting a central ulceration. Out of these ten lesions, four were on the lesser curvature, two in fundus, three in antrum and one in gastric body.

The other five lesions were not visualized on upper GI endoscopy. Three of them were purely exofitic growing and the other two were originated from small bowel. Those lesions were diagnosed by abdominal CT scan (three lesions) and as incidental findings during upper GI surgery (two lesions). Echoendoscopy and biopsy were performed on three cases, being conclusive in only one of them. 

Nine patients underwent abdominal operations before GIST resections. Only one of them had a major procedure as a treatment of severe acute pancreatitis. Those interventions did not have any impact on the laparoscopic approach. 

**Figure 1:**
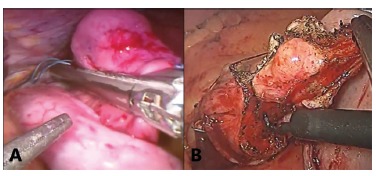
- A) Posterior gastric wall accessed through the anterior gastric wall with GIST wedge resection using mechanical laparoscopic sutures; B) anterior gastric wall GIST wedge-resected using monopolar energy followed by gastrorrhaphy

All patients underwent laparoscopic resection. Two distal gastrectomies, eleven wedge resections and two segmental small bowel resections were performed. Mechanical stapling devices were used on the majority of cases, but for six the procedures were accomplished by opening the gastric wall with ultrasonic scalpel, resecting the tumors and closing it with absorbable running sutures (Figure 1). There were no conversions. Mean operative time was 1 h and 29 min±92 (40-420 min).

**Figure 2:**
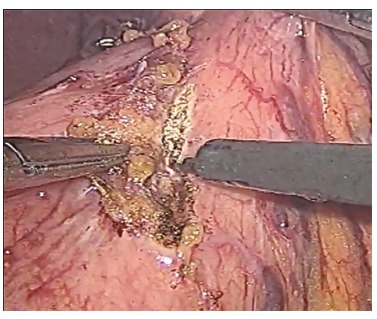
- Surgical margin resection after confirmed positive margin

All frozen surgical margins were negatives except one of the cases as frozen sections was not available at the time of surgery and it was not performed. Paraffin histology on that last case showed positive margins and patient was brought to the operation room six weeks later for margin extension still by laparoscopy (Figure 2).

The predominat histology type was the fusiform, observed on 14 patients (93%), followed by the epithelioid in only one patient (7%). 

Immunohistochemistry pannel analysis showed positive CD117 and CD34 on 14 patients (93%). Ki67 marker was positive on eight patients (53%) and desmin was seen on five (33%).

The mean lenght of hospital stay was three days (2-6). There were no need for early surgical reinterventios, no leakages nor post-operative bleeding. One of the procedures took 7 h and the same patient stayed longer in hospital secondary to prolonged postoperative ileus. Excluding a single patient reoperated for positive margins, there was no postoperative complications . 

Table 3 list patients according to prognosis classification.

**Table 3:** - Recurrence risk or death related according to tumor characteristics

**Patient**	**HPF mitosis (n)**	**Tumor size (cm)**	**Location**	**Risk**
1	0	< 2	stomach	Very Low
2	0	> 5	stomach	Low
3	0	< 2	stomach	Very Low
4	5	2 a 5	stomach	Low
5	0	2 a 5	stomach	Low
6	2	2 a 5	stomach	Low
7	0	< 2	stomach	Very Low
8	0	2 a 5	ileum	Low
9	5	2 a 5	stomach	Low
10	5	> 5	stomach	Low
11	10	2 a 5	stomach	Moderate
12	5	2 a 5	stomach	Low
13	5	> 5	stomach	Low
14	> 50	2 a 5	stomach	Moderate
15	5	2 a 5	jejunum	Low

HPF=high power field

Mean follow-up was 38 month±17(6-60 months). At the present time three patients are undergoing adjuvant imatinib therapy. Two of them for moderate recurrence risk and one for early recurrence of the disease.

## DISCUSSION

Gastrointestinal stromal tumors are complex from its diagnosis to treatment. The patient's baseline characteristics, and surgical specimen immunohistochemistry patterns (CD117 and CD34) are comparable to Brazilian and world literature^14^^,^ 23. Around 5% of GISTs are expected to be CD117 negative. Was observed that incidence in this series (n=1, 7%).

Patients are usually asymptomatic or present vague symptoms^4^^,^ 23.Therefore GISTs are often diagnosed incidentally^23^.

According to guidelines of the European Society for Medical Oncology (ESMO) tumors larger than 2 cm should be removed. Lesions under 2 cm should be followed periodically by upper GI endoscopy and biopsy; resection must be done if tumor growth is observed^3^. In this series, few patients were evaluated by echoendoscopy due to its difficult access by the time the patients were attended. 

Histopathologic evaluation of GISTs usually shows predominant fusiform type (70%). The incidence of epithelioid and the mixed types was 20% and 10 % respectivally^13^^,^ 23. Cytological analysis of the surgical specimens demonstrated fusiform or spindle cell predominance (93%). 

GIST treatment could be achieved through a multi-modalities approach, but surgical resection remains the primary focus^27^. Target therapy or even radiotherapy can be used in some exceptional cases and multiple procedures maybe needed for recurrent lesions.

Laparoscopic primary GIST resection should be considered by surgeons with advanced laparoscopic skills^7^^,^ 21. Laparoscopic GIST resection has the advantages over the open conventional approaches as being more precise procedure and results, with better postoperative and immunological outcome, but these findings still need to be confirmed. 

A great variability among laparoscopic techniques used in the present study correlates with the disease heterogeneous nature. Procedures ranged from mechanical stapling wedge resections to distal gastrectomies. Oncology principles can be efficiently respected in the laparoscopic approach and larger lesions (>5 cm) as well. Three patients in the study had large lesions. Free surgical margins should be the treatment goal confirmed by the frozen sections. Positive margins should be removed at the primary resection or during reoperation if diagnosis was performed on paraffin sections.

Tumor capsule should be preserved in order to avoid trocars seeding, and direct contact with the lesion should also be avoided. Reoperations for margin resection can be done laparoscopically as was done in one of these cases.

Han (2012) considers tumor capsule violation as a worsening prognostic factor. When it occurs, patients should be treated with routine adjuvant therapy 9 ^,^ 10.

Comparable results were published by other authors^5^^,^ 19 ^,^ 20 ^,^ 25. Novitsky (2006) reported 50 laparoscopic resections. Average tumor size was 4 cm and 8% recurrence rate (mainly hepatic metastasis) was observed over a 36 months follow-up^25^. Similar to these findings, his patients did not develop local recurrence or trocar site metastasis. The author considers the laparoscopic approach superior for GIST surgical treatment, especially for gastric lesions. This opinion is shared by our group.

Among prognostic criteria, mitosis index is the most related to recurrence^2^^,^ 22. In these authors experience, patient with higher number of mitosis per 50 HPF analysis suffered early disease recurrence. 

Continued adjuvant chemotherapy use on high risk GIST patients without documented local recurrence or metastasis is still being a matter of discussion^6^^,^ 8 ^,^ 11 ^,^ 18. Some studies showed efficacy of this practice with disease free interval increase^6^^,^ 11. This choice has to be considered by the medical oncologist and the high risk GIST patient.

## CONCLUSION

Laparoscopic GISTs resection for primary gastric and intestinal lesions seems to be feasible and safe. Comparative studies from larger series of patients are needed to evaluate more advantages of laparoscopic approaches..
